# Development of a qPCR assay for *Fasciola* spp. identification and a deep amplicon sequencing method for differentiation of fluke species in UK livestock

**DOI:** 10.1371/journal.pntd.0014006

**Published:** 2026-02-17

**Authors:** Muhammad Abbas, Kezia Kozel, Olukayode Daramola, Nick Selemetas, Qasim Ali, Shoaib Ashraf, Ibrahim Isah, Inaki Deza-Cruz, Sai Fingerhood, Mark W. Robinson, Eric R. Morgan, Umer Chaudhry, Martha Betson

**Affiliations:** 1 Discipline of Comparative Biomedical Sciences, School of Veterinary Medicine, University of Surrey, Guildford, United Kingdom; 2 School of Veterinary Medicine, University of Lancashire, Preston, United Kingdom; 3 Discipline of Microbes, Infection and Immunity, School of Veterinary Biosciences, University of Surrey, Guildford, United Kingdom; 4 Department of Pathobiology, University of Veterinary and Animal Sciences, Swat, Pakistan; 5 Department of Pathobiology, College of Veterinary Medicine, Riphah International University, Lahore, Pakistan; 6 Department of Biomedical Sciences, Ross University, School of Veterinary Medicine (RUSVM), St Kitts and Nevis, West Indies; 7 Department of Veterinary Parasitology and Entomology, Faculty of Veterinary Medicine, Ahmadu Bello University, Zaria, Nigeria; 8 The Royal (Dick) School of Veterinary Studies and The Roslin Institute, The University of Edinburgh Easter Bush Veterinary Centre, Midlothian, Edinburgh, United Kingdom; 9 Department of Veterinary Pathology, University of Nottingham, Nottingham, United Kingdom; 10 School of Biological Sciences, Queen’s University, Belfast, United Kingdom; 11 Department of Veterinary Biomedical Sciences, Lewyt College of Veterinary Medicine, Long Island University, Brookville, New York, United States of America; University of Liverpool, UNITED KINGDOM OF GREAT BRITAIN AND NORTHERN IRELAND

## Abstract

**Background:**

Trematode parasites, or flukes, are a significant economic threat to ruminant production worldwide. Traditional diagnostic methods rely on egg sedimentation from faeces, a time-consuming methodology lacking sensitivity and specificity. This study aimed to develop and validate two detection methods: firstly, qPCR for accurate identification of *Fasciola* spp., and secondly, a deep amplicon sequencing technique for identifying fluke species using faecal sedimented egg DNA.

**Methodology:**

To detect *Fasciola* spp., infection, primers targeting mitochondrial DNA were repurposed to develop a SYBR Green qPCR assay. For the identification of fluke species, a deep amplicon sequencing approach was developed. A reference sequence library and taxonomy file were generated for 21 fluke species, potentially enabling species-level sequence read separation for a range of trematodes and extraction of amplicon sequence variants (ASVs). To validate the qPCR and deep amplicon sequencing approach, 402 faecal samples were collected from cattle and sheep across the UK. Fluke eggs were isolated by sedimentation, screened by microscopy and qPCR, Sanger sequencing and deep amplicon sequencing to identify fluke eggs to species level.

**Results:**

qPCR demonstrated high analytical sensitivity, detecting *Fasciola hepatica* DNA down to 19.2 fg and *F. gigantica* down to 6.4 fg, with no cross-amplification of other flukes. Deep amplicon sequencing was able to detect as few as five *F. hepatica* and *Calicophoron daubneyi* eggs and identify mixed infections. High levels of co-infection (14.4%) of *F. hepatica* and *C. daubneyi* were observed in faecal samples, followed by single infections with *C. daubneyi* (12.6%) and *F. hepatica* (3.2%). Notably, deep amplicon sequencing detected *F. hepatica* in 20 samples missed by qPCR. Data analysis identified 55 and 32 ASVs for *F. hepatica* and *C. daubneyi*, respectively, with phylogenetic clustering within their respective clades.

**Conclusion:**

This study developed a qPCR assay for *Fasciola* spp. detection and validated a deep amplicon sequencing for fluke species differentiation. These approaches are able to identify fluke species in excreta from infected ruminants and provide additional valuable tools for enhancing fasciolosis surveillance and control.

## Author summary

Flukes are flatworm parasites that can cause disease in domestic and wild animals and humans. The main species infecting cattle and sheep globally are the liver fluke *F. hepatica* and *F. gigantica*, with other species emerging, including *Calicophoron daubneyi,* the predominant rumen fluke species in Europe. *Fasciola* spp. infections result in serious economic losses. The traditional method of diagnosing fluke infection involves observation of eggs in faecal samples under the microscope, but this can be time-consuming and error prone, since the eggs of different species often look similar. In this study, we developed and validated two methods to improve detection: qPCR, a sensitive DNA-based test to identify *Fasciola* infections, and a DNA deep amplicon sequencing technique that can accurately differentiate between different fluke species, for example, *F. hepatica* and *C. daubneyi* species. We tested these methods using faecal samples collected from cattle and sheep across the UK. The qPCR could detect small amounts of *Fasciola* DNA, while deep amplicon sequencing was more sensitive, identifying different fluke species from as few as five eggs. Our study found that co-infections of *F. hepatica* and *C. daubneyi* are common in the UK. The approaches we have developed could be valuable tools for improving fluke diagnosis and enabling better control of this important parasitic disease.

## Introduction

Trematode parasites, or flukes, are widespread globally and include several species that cause serious disease in animals and humans. Fasciolosis is a neglected foodborne tropical disease caused by the zoonotic flukes *Fasciola hepatica* and *Fasciola gigantica*. Unlike other neglected tropical diseases, *Fasciola* infections in humans and animals have a broad reach globally, being found in more than 75 countries, with 2.4 million people infected, and millions more at risk [1]. The prevalence in livestock is less well known; however, a recent meta-analysis suggests the global prevalence of fasciolosis in cattle and sheep across continents ranges from 12-97% and 9–58% respectively [2]. *F. hepatica* is widely distributed in temperate regions, including the UK, Europe, South America, and parts of Oceania [3–7], and *F. gigantica* predominates in tropical and subtropical areas of Africa and Asia [8,9]. The rumen fluke *Calicophoron daubneyi* is an emerging parasite of ruminants in western Europe, reported in a range of countries including Ireland, UK, Belgium, France, Germany, Spain and Italy [10–17]. *C. daubneyi* belongs to the family Paramphistomidae, a group of flukes typically found in the forestomachs of ruminants. Unlike the flattened morphology common to most trematodes, these flukes exhibit a distinct conical shape as adults [10]. Co-infections with *F. hepatica* have been found in the UK, France, and Germany [11–13]. Recent studies suggest that paramphistomosis prevalence is now substantially increasing in certain regions of the UK and in Europe [9–11]. However, currently, diagnostic options for rumen fluke are limited and need further research. Futhermore, in endemic settings, co-infections and limited access to advanced diagnostics may complicate surveillance and control [10,14]. Molecular tools capable of species-level identification of fluke in faecal samples could provide a valuable addition to diagnostic capacity in both veterinary and public health settings.

Ensuring food security is increasingly challenging with a growing global population. In 2020, the agri-food sector contributed 115 billion GBP, making up 6.0% of the UK economy [15], with other national economies considerably more dependent on farming. Recent global estimates indicate that fasciolosis may cost annual losses in animal productivity exceeding US$3.2 billion [16,17]. In the UK, fasciolosis prevails in ruminants, costing the cattle industry 13–40 million GBP yearly, dairy farm net profits by 12% and beef farm by 6% [18]. *Fasciola* infections can lead to delayed animal slaughter [19] and condemnation of damaged livers [20]. According to the Food Standards Agency (FSA), UK in 2014, 22% of British cattle livers and in 2015, 16.5% of cattle livers were condemned due to fluke [21,22]. Losses to fasciolosis are widespread; for example, Australia faces one of the highest disease burdens, with estimated annual losses reaching approximately 129 million (range 38–193 million) AUD annually [7]. Infected animals suffer reduced weight, anaemia, reduced milk yield and fat content [20], lower reproduction, and higher mortality [23].

Rumen flukes such as *C. daubneyi* may also impact livestock productivity, including growth rates, fertility, meat quality, wool and milk production, and may occasionally cause mortality [10]. This is particularly associated with the larval stages of rumen fluke, which are released into the duodenum, where they attach to the intestinal lining and cause tissue damage. Although chronic infection with *C. daubneyi* adult stages is not typically associated with clinical disease [24], the parasite can alter rumen fermentation kinetics [25]. However, the overall impact on productivity is not yet fully resolved [10].

Traditionally, diagnosis of both *Fasciola* and *Calicophoron* infections relies on microscopic identification of fluke eggs in the host faeces [26–28], with eggs usually observed 10 − 12 weeks post-infection and thereafter [27–29]. The effectiveness of microscopy relies on personnel training and expertise. Moreover, it becomes labour-intensive when handling a large number of samples, particularly if the person lacks sufficient experience, leading to low sensitivity [30]. Distinguishing between *F. hepatica* and *C. daubneyi* solely based on egg morphology in faecal samples can be challenging as both parasites produce eggs with comparable sizes and shapes [12,31]. Although, it is reported that *F. hepatica* eggs can be identified by their operculum [32] and yellowish colour [33], these features can be difficult to observe consistently using standard light microscopy. As a result, relying solely on egg morphology for diagnosis may lead to misidentification. However, a new fluke egg count technology has recently been developed, integrating digital imaging and artificial intelligence to automate fluke egg detection and species differentiation using a modified FECPAKG2 protocol [34]. Further, molecular diagnostics based on *Fasciola* DNA detection are rapidly progressing [35]; for instance, qPCR [36] and PCR techniques have been applied to adult worms and infected snails [37] and eggs sedimented from faeces [3]. Additionally, techniques such as nested PCR [27] and Loop-Mediated Isothermal Amplification (LAMP) are being explored [38] and assessed for their speed, reliability, and accuracy compared to other methods [38,39]. However, significant challenges remain in detecting *Fasciola* DNA in faecal material, highlighting the need for a reliable, cost-effective, time-efficient and accurate diagnostic method, which can handle medium to large sample sizes and is capable of differentiating *F. hepatica* infections from other fluke species.

Next-generation sequencing technologies are transforming the diagnosis of infectious diseases whilst also paving the way for new research areas, such as microbiome studies [40–42]. Amplicon sequencing using next-generation approaches has been applied to identify gastrointestinal nematode species in ruminants (“nemabiome”) [43], equines [44,45], canids [46] and in pigs [47]. Further, this method was used to quantify trypanosome and piroplasm species in ruminant blood samples (“haemoprotobiome”) [48,49], and has been applied to quantify single species adult fluke infections in ruminants [8,50,51]. In a recent study, next-generation amplicon sequencing proved superior to traditional Sanger sequencing for the identification of *Fasciola* species, using adult worms, faecal samples, and mock mixtures of DNA from both species [52]. Mixed fluke infections (e.g., *F. hepatica* and *C. daubneyi*) in ruminants are emerging [8,50], however, next-generation amplicon sequencing has not yet been used to understand fluke egg communities in single and mixed species infections of liver and rumen fluke in material sedimented from faeces of naturally infected animals.

In this study, we hypothesised that (i) to develop a cost-effective real-time diagnostic tool, a SYBR Green-based qPCR assay targeting the mitochondrial NADH1 (mt-ND1) DNA could provide a sensitive and specific method for *Fasciola* spp. detection in egg DNA from sedimented faeces of naturally infected ruminants, and (ii) a deep amplicon sequencing method could accurately differentiate between fluke species such as *Fasciola* spp. and *C. daubneyi*, and identify mixed infections. We chose to develop a SYBR Green qPCR-based assay rather than an assay using probe-based chemistries (e.g., TaqMan) due to its cost-effectiveness, simplicity and accessibility, making it useful for diagnostic applications in resource limited settings where fasciolosis is endemic. To develop the deep amplicon sequencing method, we combined two previously published approaches developed for adult flukes of *Fasciola* and *Calicophoron* spp. [8,51] and created an assay capable of differentiating between various species of fluke. Both methods were validated using field samples of fluke eggs and adults collected from different regions across the UK. In parallel, we applied microscopy, PCR, and Sanger sequencing to the same samples and found that each detection method has strengths and limitations in detecting fluke infections and differentiating between fluke species.

## Materials and methods

### Ethical statement

Non-invasive collection of faecal samples was approved by the NASPA (Non-Animal Scientific Procedures Act) sub-committee of AWERB, University of Surrey, UK, under the reference NASPA-2122-04 for the project “Developing Novel Rapid Diagnostics for Neglected Parasitic Diseases.” Adult flukes were collected at licenced slaughterhouses and through post-mortem examination. Completion of a University of Surrey SAGE-AR (ID 638929-638920-101535552) indicated that no formal ethical approval was required for adult fluke sampling.

### Positive control samples and DNA extraction

All adult *F. hepatica* worms and sedimented eggs were collected from UK, whilst adult worms and eggs purified from adult worms of *F. gigantica*, *C. daubneyi*, *Paramphistomum epiclitum,* and *Explanatum explanatum* were collected from abattoirs in Pakistan in our previous studies [8,50,51,53,54]. Further, DNA extracted from *Teladorsagia circumcincta* from UK sheep was provided by Dr Neil Sargison University of Edinburgh [55]. Adult fluke worms were identified through morphological assessment, then washed five times with sterile phosphate buffer saline (PBS). A small piece of head tissue (excluding egg contamination) was sectioned and subjected to three washes in PBS following DNA extraction using a previously described protocol [50] using the DNeasy Blood & Tissue Kit (Qiagen, USA).

DNA was extracted from sedimented eggs of positive controls (*F. hepatica*, *F. gigantica*, *C. daubneyi*, *E. explanatum*) and from faecal sedimented material from field samples utilising the DNeasy PowerSoil Pro Kits (Qiagen, USA), with slight modification. Briefly, these modifications included incubating the sedimented material with the lysis buffer (CD1) for 10 minutes at 65°C, followed by 10 minutes of bead-beating with 1-minute incubations on ice every 3 minutes to minimise DNA shearing using a TissueLyser LT (Qiagen, USA). The manufacturer’s protocol was then followed, with DNA being eluted in 10mM Tris buffer and stored at -80°C for downstream analysis. The success of DNA extraction (i.e., presence of DNA) was confirmed using a BioDrop reader (Biochrom, UK) and Qubit dsDNA HS and BR Assay Kits (Invitrogen).

### Field sample collection

To engage cattle and sheep farmers, the study was advertised by email to registered veterinary practitioners using the Royal College of Veterinary Surgeons Find a Vet site filtering for practices specialising in cattle, sheep and/or goats, and camelids. In addition, societies for sheep and cattle listed by the Department for Environment, Food and Rural Affairs (https://www.gov.uk/government/publications/lists-of-recognised-animal-breeding-organisations) in the UK were also approached via the email contact listed. Participants were sent a Royal Mail prepaid SafeBox with a sampling kit, a short questionnaire, a participant information sheet and consent form according to ethical requirements. No samples were used without the written informed consent of the farmer. A total of 402 faecal samples were collected from 19 cattle and sheep farms across various geographical regions of the UK through 10 registered veterinary practitioners from December 2022 to May 2024. The field samples included both pooled and individual faecal samples, although in some cases information on pooling was not provided by the farmers. Further, there were samples with history of fluke infection, no history of fluke, and unknown history (S1 Table). The cattle and sheep sampled represented diverse age groups, ranging from calves and lambs to adults. Notably, only one sheep farm from Wales was included, and no faecal samples were collected from Northern Ireland (S1 Table).

In addition to faecal samples, adult *F. hepatica* worms were collected from abattoirs and at post-mortem examination from cattle (n = 2) originating from West and East Sussex, as well as from sheep (n = 10) from West Sussex, Kent, Derbyshire, Renfrewshire Scotland and County Tyrone Northern Ireland. All samples were transported to the School of Veterinary Medicine at the University of Surrey, UK, and subsequently stored at -20°C for further analysis. DNA from the adult flukes was extracted as described in section “Positive control samples”.

### Morphological identification of fluke eggs

Faecal samples were initially processed using standard sedimentation methodology [3] and then to streamline the process a time-saving method, Flukefinder (Diagnostic System, USA), was also used [12,56], with slight adjustments to the manufacturer’s protocol to maximise the recovery of eggs. Both methods utilised 7–10 g of faecal material (rather than the 2 g recommended by Flukefinder manufacturer) which was combined with 50 ml of water, sieved through gauze and then passed through the Flukefinder apparatus. For both methods, the collected filtered material was then mixed with 250 ml of water in a conical beaker. After three minutes, the supernatant was removed and water then topped up; this step was repeated thrice for clarity. The sediment was transferred to a 50 ml centrifuge tube filled with water, and the supernatant was aspirated after 3 minutes. This process was repeated with a 15 ml centrifuge tube. Finally, the sediment was transferred to a 1.5 ml Eppendorf tube in 1 ml of PBS and was stored at 4°C for subsequent microscopy and DNA isolation.

**Fig 1:**
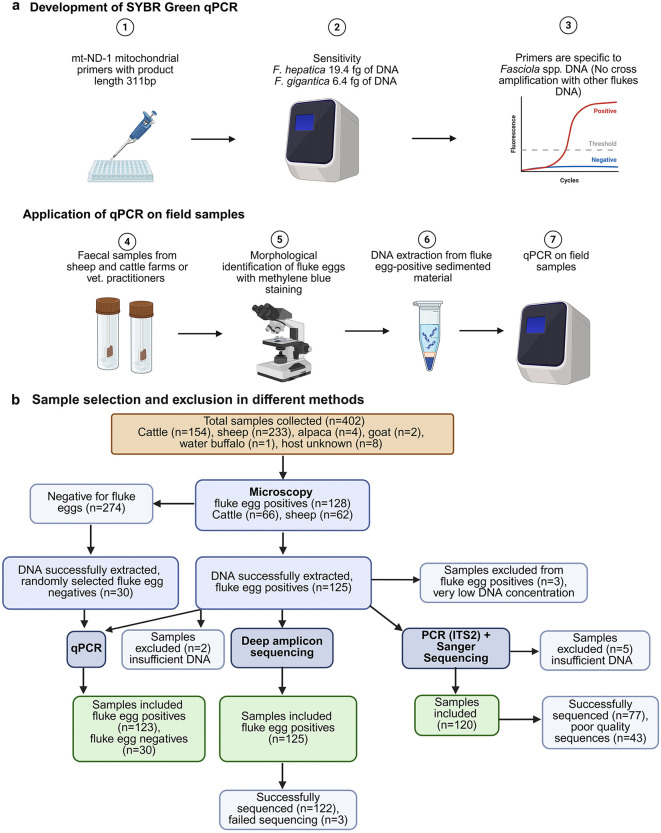
Development of qPCR and criteria for sample inclusion and exclusion. **(a)** Workflow adopted for developing qPCR and screening of faecal samples for the presence of *Fasciola* infection mainly in sheep and cattle. **(b)** Flow diagram indicating the number of samples included and excluded from testing using different fluke detection methods in this study. Samples were excluded from testing based on specific criteria, including absence of fluke eggs, failed DNA extraction, poor sequence quality, non-specific amplification, absence of sequence reads, or insufficient DNA. https://BioRender.com/dkxo0ek.

Morphological egg examination of the resuspended pellet was conducted by inspecting successive 100 μL aliquots (up to 500 μL) of the suspension stained with 0.5% methylene blue (Pro-Lab, UK) in a counting chamber (Graticules Optics Limited, UK) under a compound microscope (Nikon, Japan) at 100 × magnification with a lens containing a graticule. Once eggs were observed no further aliquots were examined. To determine total eggs counts eggs were counted in 100 μL, and the count was scaled-up to 1 mL to estimate total egg numbers. The EPG was calculated by dividing the total egg count by the starting mass of faeces. Samples positive for fluke eggs (n = 128) were assessed for the presence of *F. hepatica* or *C. daubneyi* eggs based on their morphological characteristics, including size, shape, colour, and operculum [12,31–33]. The overall workflow is summarised in Fig 1a (steps 4–5).

Any sedimented faecal samples that were identified as positive for fluke eggs by microscopy were subsequently subjected to DNA isolation utilising the DNeasy PowerSoil Pro Kits (Qiagen, USA) according to the manufacturer’s instructions as described in the section “Positive control samples”.

### Molecular identification of fluke eggs

PCR was performed using universal ITS2 primers [57] on fluke egg-positive samples and positive controls. This was followed by Sanger sequencing to confirm the presence of DNA corresponding to different fluke species.

All PCR reactions were prepared with DreamTaq Green PCR master mix (Thermo Scientific, USA) in a 25 μL reaction mix, with primer concentrations of 200 nM and 4 μL of sample DNA template. The PCR cycling conditions were initial denaturation at 95°C for 5 minutes, followed by 35 cycles of denaturation at 95°C for 1 minute, annealing at 55°C, for 1 minute, and extension at 72°C for 1 minute. The final extension step was carried out at 72°C for 5 minutes. Positive controls consisted of DNA from *F. hepatica* and *F. gigantica* adult worms. The resulting PCR products were purified and cleaned using a NucleoMag kit for clean-up and size selection of NGS library prep reactions (MACHEREY-NAGEL, GmbH & Co.KG). Sanger sequencing of the PCR product was performed by Source Biosciences, UK and Eurofins Genomics, Germany. All obtained sequences were visualised using Geneious version 8.0.5 (https://www.geneious.com), and the FASTA sequences were submitted to the BLASTn tool with ≥98% identity to available sequences hosted by NCBI to confirm fluke species identity.

### Development and validation of a SYBR green qPCR to detect *Fasciola* eggs at species level

A SYBR green qPCR assay was developed to detect *Fasciola* spp., using mt-ND1 primers with an expected amplicon size of 311 bp (S3 Table). These mt-ND1 primers were previously designed and employed in a meta-barcoded PCR [50]. In the SYBR green assay, 3 μL of DNA was subjected to qPCR using 10 μL of 2X SsoAdvanced Universal SYBR Green Supermix (Bio-Rad, USA), resulting in a total reaction mix volume of 20 μL, including 500 nM of mt-ND1 primers.

The cycling program was initial denaturation at 98°C for 3 mins, followed by 40 cycles of denaturation at 98°C for 15 secs and annealing at 60°C for 30 secs on a CFX96 Real-Time PCR machine (Bio-Rad, USA). Subsequently, a melt curve was generated from 65°C to 95°C with an increment of 0.5°C for 0.05 secs per plate read. Positive controls were employed as described above, and molecular-grade water was used as no-template controls to monitor for potential contamination in the qPCR runs. No amplification was observed in the no-template control well in any of the qPCR runs. All samples were subjected to qPCR in triplicate, and the resulting data was visualised using CFX Maestro Version 5.3.022.1030 (Bio-Rad, USA). Positivity was confirmed by checking for a melt peak at 81.5°C and 82°C for *F. hepatica* and *F. gigantica*, respectively. PCR setup and post-PCR processes were conducted in separate PCR hoods and laboratory areas using dedicated pipettes and filtered tips.

Detection sensitivity limits of the assay were assessed using five-fold serial dilutions of *F. hepatica* (ranging from 300 pg to 0.768 fg) and *F. gigantica* (ranging from 500 pg to 1.28 fg) adult worm DNA. The sensitivity of the qPCR assay was validated using DNA extracted from adult *F. hepatica* and *F. gigantica* flukes, whose species identification was confirmed based on morphology and ITS2 Sanger sequencing, which served as positive reference standards. DNA was quantified using Qubit dsDNA HS and BR Assay Kits (Invitrogen) prior to the qPCR sensitivity assay to ensure accurate and consistent DNA input. To remove background noise, Ct thresholds were set between 100–200 RFU. The analytical specificity of the assay for *Fasciola* spp. was evaluated by testing one ng of DNA from other prevalent flukes and nematodes found in sheep and cattle. These included *C. daubneyi*, *P. epiclitum*, *E. explanatum*, and the nematode *Teladorsagia circumcincta.* The sensitivity and specificity tests were conducted in triplicate and repeated twice.

The reliability of the method was measured by comparing inter- and intra-assay variations in Cq values for *F. hepatica* and *F. gigantica* DNA. For validation, the newly developed qPCR assay was applied to DNA extracted from sedimented material from all faecal samples which were fluke-egg positive by microscopy, and a subset of 30 randomly selected fluke egg-negative samples to assess the sensitivity of the qPCR assay.

### Development and validation of deep amplicon sequencing to detect fluke eggs at species level

A universal ITS2 rDNA marker for 21 different fluke species (S2 Table; https://data.mendeley.com/datasets/zyvwc6ppy8/2) was used, targeting coding regions of 5.8S and 28S rDNA and expected to produce a fragment of 490–743 bp [57]. The primers were meta-barcoded by adding Illumina adaptor sequences to both forward and reverse primers, along with up to three random ‘N’ nucleotides positioned between the adaptor sequences and the locus-specific primers. Additionally, modified phosphate bonds were added between the last three nucleotides of each primer to enhance their stability (S3 Table) and used in PCR amplification to detect fluke species. To assess the representation of species read depth, mock DNA mixtures using eggs obtained from adult worms as described in previous studies [8,50,51,53,54]. The species of the eggs was confirmed by extracting DNA from a sample of eggs and conducting ITS2 PCR followed by Sanger sequencing as described above. Mock mixes were prepared in triplicate by pooling 250 eggs of each species, including *F. hepatica*, *F. gigantica* and *C. daubneyi*, and subjected to amplicon sequencing in triplicate. PCR cycle numbers were adjusted to 35 ×, 30× and 25× in the first round to examine their effect on species representation. Further, to evaluate species representation using egg DNA, we created seven mock egg pools in triplicate, adjusting the proportion of *F. hepatica* and *C. daubneyi* in an approximate total of 250 eggs with ratios of 99:1, 90:10, 70:30, 50:50, 30:70, 10:90, and 1:99. Moreover, to test the threshold of deep amplicon sequencing, pools of equal egg proportions (50 eggs) were prepared from three out of four species (*F. hepatica*, *F. gigantica*, *E. explanatum*, *C. daubneyi*). The fourth species (*F. hepatica* or *C. daubneyi*) was then added in decreasing numbers of 500, 50, 20, 15, 5, and 0 eggs, creating six mock pools. The first round of PCR was performed using the KAPA HiFi PCR Kit (KAPA BIOSYSTEMS, South Africa). The modified primer sets, adaptors, barcoded PCR amplification conditions, magnetic bead purification methods, and bioinformatic analysis were based on our previously described methods [50]. The first-round PCR products were subjected to a second-round PCR using a barcoded primer set to attach a unique barcode index fragment required for Illumina sequencing [48].

Second-round barcoded PCR products 10 μL of each were combined to create a pooled library, and then purified by agarose gel electrophoresis to remove non-specific products and adaptor dimers. During post-run processing, the MiSeq system separated all sequencing data by sample quality using the barcoded indices to generate FASTQ files (raw sequence read files available at https://data.mendeley.com/datasets/zyvwc6ppy8/2). Deep amplicon sequencing statistics are provided for all samples (S4 Table).

The FASTQ files obtained from the post-run Illumina MiSeq (BioProject ID PRJNA1273189) were analysed in Mothur/1.41.0-Python-2.7.15 [58] using the High-Performance Computing (HPC) cluster at the University of Surrey, UK. Pipelines described in our previous study [59] were utilised with modifications of the newly developed reference sequence library (script available at https://data.mendeley.com/datasets/zyvwc6ppy8/2).

To generate a taxonomy file, ITS2 rDNA reference sequences (n = 545) were obtained from NCBI, representing 21 fluke species (S2 Table). The genetic distances between different fluke species were then calculated based on the sequenced region. Following the extraction of the taxonomy file, quality filtering was conducted for the identification of unique fluke sequences, detailed count tables were generated and an alignment (ALIGN) file of sequences across all samples was produced. This workflow ensured a robust, high-quality dataset suitable for downstream taxonomic studies for flukes using a series of commands (https://data.mendeley.com/datasets/zyvwc6ppy8/2).

Firstly, the R script was applied to count table and alignment (ALIGN) file. The script started by cleaning the sequences, trimming whitespace, and converting them to lowercase to ensure consistency. The sequencing read data was filtered to remove sequences of less than 200 bp in length, ensuring that only high-quality sequence reads remained. A custom function was employed to write the sequences into a combined FASTA file, preserving both the sequence names and read counts. Next, the script utilised the Biostrings package to clean the sequences by removing ‘N’ characters and ambiguous bases, saving the high-quality sequences to a final FASTA file (R script available at https://data.mendeley.com/datasets/zyvwc6ppy8/2). This comprehensive approach ensured accurate sequence extraction for subsequent analysis. Finally, the sample-wise separated FASTA files were subjected to remote NCBI BLASTn loop command using “blastn: 2.16.0+” in the HPC cluster system (command line available https://data.mendeley.com/datasets/zyvwc6ppy8/2).

Sequences were then blasted against the NCBI database and results deposited in a Mendeley data base (BLASTn results available at https://data.mendeley.com/datasets/zyvwc6ppy8/2). Next, the count table and alignment (ALIGN) file were further used for the extraction of amplicon sequence variants (ASVs)**.** The R script extracted specific ASVs corresponding to the target species of flukes, applying cutoff values of 250 reads each (NCBI accession numbers; PV752375-PV752429, PV752431-PV752462).

### Statistical analysis

For qPCR analysis, the raw Cq values were extracted from CFX Maestro Version: 5.3.022.1030 (Bio-Rad, USA), and a linear standard curve was created by plotting DNA quantities against the average Cq values for each concentration tested. For deep amplicon sequence read data analysis, the sequences from the bioinformatics pipeline were further analysed for sequence accuracy and percentage identity using remote blastn: 2.16.0+ with the NCBI database. To determine the percentage composition of *F. hepatica*, *F. gigantica*, *C. daubneyi*, and *E. explanatum* in mock egg mixtures (positive control) and field samples, species composition percentages were calculated by dividing the classified sequence reads for each species by the total quality-filtered reads per sample. A one-way ANOVA was applied to assess the sequence representation of mock mixes comprising different fluke species eggs across varying PCR amplification cycles. The association between qPCR and deep amplicon sequencing results was assessed using the Fisher exact test. The R packages dplyr [60], ggplot2 [61] and the ggpubr [62] were used to determine the correlation between fluke egg counts (FEC) and Cq values, undertake regression analysis and generate a scatterplot. Receiver operating characteristic (ROC) curves, confusion matrices and Cohen’s kappa values were calculated using R version 4.3.3 with packages pROC [63], caret [64] and irr [65], respectively. Mean and standard deviation were calculated in R using base functions (mean, sd) and coefficient of variation using the DescTools package [66]. All visualisations of data were performed in R version 4.3.3 using packages available on the R Project website (https://www.R-project.org/), and all R scripts are available at https://data.mendeley.com/datasets/zyvwc6ppy8/2

### Phylogenetic analysis

Phylogenetic trees were generated from unique ITS2 rDNA reference sequences from 21 different fluke species downloaded from NCBI GenBank (S2 Table). The sequences were aligned using the MUSCLE alignment tool in Geneious v8.0.5 (Biomatters Ltd, New Zealand) and genetic distances were calculated (S5 Table). Further, a phylogenetic tree of the unique ITS2 rDNA sequences for all 21 fluke species and ASVs of the flukes was constructed using the Neighbor-Joining method [67]. The evolutionary distances were computed using the Maximum Composite Likelihood method [68] in MEGA11 [69] with a bootstrap value of 2000 [70].

## Results

### Fluke identification by microscopy

A total of 402 faecal samples were examined, out of which 128 (31.84%) were positive for fluke eggs. The sampled animals included cattle (n = 154), sheep (n = 233), water buffalo (n = 1), alpaca (n = 4), and goats (n = 2), with animal species unspecified for 8 samples. Of these samples, 191 (47.51%) had a history of *Fasciola* infection, 119 (29.6%) had no history, and 92 (22.88%) had an unknown history (S1 Table). Based on egg morphology and staining, 30 (23.43%) samples were identified as *C. daubneyi*, 71 (55.46%) as *F. hepatica*, five (3.9%) as mixed infections (*C. daubneyi* and *F. hepatica*), and 22 (17.18%) remained unconfirmed (S6 Table).

### PCR followed by Sanger sequencing of the fluke identified by microscopy

Out of 128 samples positive for fluke eggs, DNA was successfully extracted from 125 (97.65%). PCR was performed on 120 samples using universal ITS2 primers, followed by Sanger sequencing (Fig 1b, S6 Table). There was insufficient DNA for 5 samples to perform the ITS2 PCR. From the 71 samples believed to be *F. hepatica* by microscopy (EPG range: 1–315, standard error (SE): 5.87), Sanger sequencing confirmed seven as *F. hepatica* (EPG range: 2–100, SE: 13.42), 34 as *C. daubneyi* (EPG range: 1–170, SE: 7.6) and 23 samples (EPG range: 1–315, SE: 13.32) demonstrated poor sequence quality. Of the 30 samples identified as *C. daubneyi* by microscopy (EPG range: 1–60, SE: 2.27), 18 were confirmed as *C. daubneyi* (EPG range: 2–60, SE: 3.18), one was confirmed to be *F. hepatica* (EPG:28), one was identified as *Paramphistomum epiclitum* (EPG: 3), and ten (EPG range: 1–30, SE: 2.77) demonstrated poor sequence quality by Sanger sequencing. Of the five samples which were identified as mixed infections by microscopy (*F. hepatica* and *C. daubneyi*) (EPG range: 28–160, SE: 23.61), Sanger sequencing identified one sample as *F. hepatica* (EPG: 121), three as *C. daubneyi* (EPG range: 52–160, SE: 40.59), and sequence quality was poor for one sample (EPG: 88). In the 22 samples where microscopy could not determine the fluke species present (EPG range: 1–420, SE: 18.89), two samples were confirmed as *F. hepatica* (EPG range: 2–14, SE: 6), 10 as *C. daubneyi* (EPG range: 1–420, SE: 41.61), and nine demonstrated poor sequence quality (EPG range: 1–10, SE: 1.15) (Table 1, S6 Table).

**Table 1:** Fluke species identification by microscopy, ITS2 PCR, and Sanger sequencing on fluke egg-positive samples (n = 128).

Microscopy positive	PCR					Sanger sequencing using ITS2						
	Negative	Positive	Not performed	ND^2^	Total	*Fasciola hepatica*	*Calicophoron daubneyi*	*Paramphistomum epiclitum*	PSQ	Not performed	ND^2^	Total
*Fasciola hepatica*	10	54	5	2	71	7	34	0	23	5	2	71
*Calicophoron daubneyi*	4	26	0	0	30	1	18	1	10	0	0	30
Mixed	0	5	0	0	5	1	3	0	1	0	0	5
Fluke^1^	3	18	0	1	22	2	10	0	9	0	1	22
Total	17	103	5	3	128	11	65	1	43	5	3	128

^1^Unidentified flukes; ^2^ND = DNA extraction failed; PSQ = poor sequence quality.

**Fig 2:**
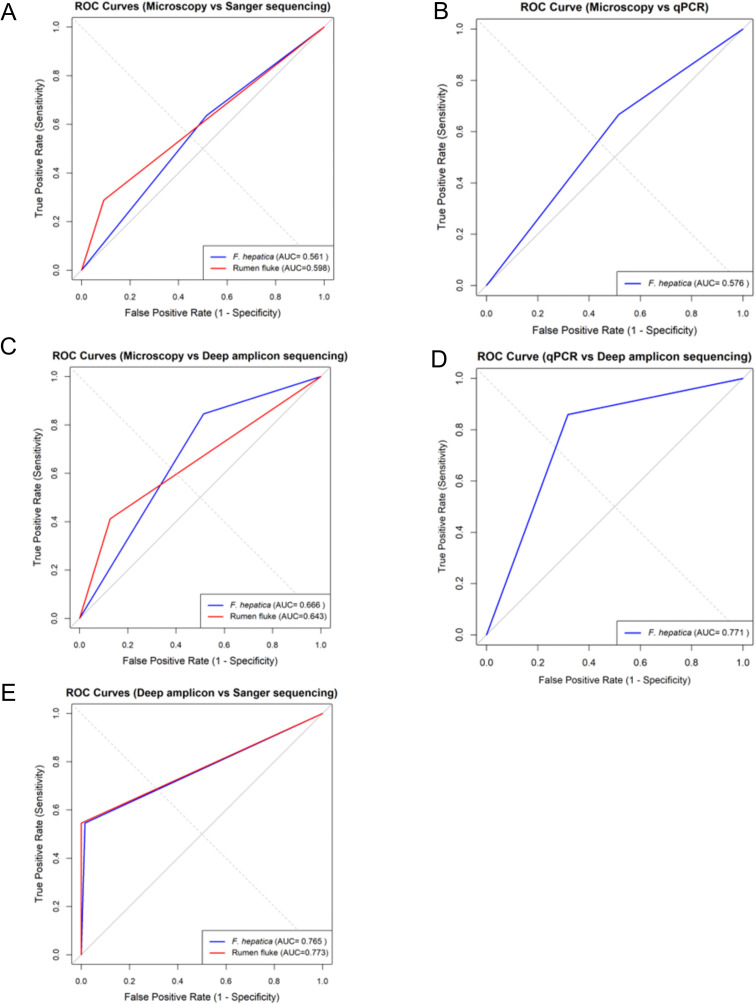
Receiver Operating Characteristic (ROC) curves to investigate agreement between microscopy, qPCR, Sanger sequencing, and deep amplicon sequencing for identification of fluke species in microscopy positive faecal samples. Each panel shows the ROC curve comparing two methods (A) microscopy and Sanger sequencing (B) microscopy and qPCR, (C) microscopy and deep amplicon sequencing, (D) qPCR and deep amplicon sequencing, (E) deep amplicon sequencing and Sanger sequencing. Blue curves represent *F. hepatica* and red curves represent rumen flukes (*C. daubneyi* + *P. epiclitum*). The area under the curve (AUC) values are displayed in the legends. The x-axis represents the false positive rate (1 – specificity) and the y-axis represents the true positive rate (sensitivity). A diagonal dashed line indicates random classification performance (AUC = 0.5).

ROC curve analysis showed poor agreement between microscopy and Sanger sequencing in the identification of *F. hepatica* (AUC = 0.56, 95% CI: 0.40–0.72) and rumen fluke (AUC = 0.59, 95% CI: 0.49–0.70). AUC values were not determined for samples that were classified as mixed infections by microscopy or where fluke species could not be identified by microscopy because Sanger sequencing of PCR amplicons cannot detect mixed infections [71]. Cohen’s kappa value was 0.05 (Z = 1.153, p = 0.24), showing very low and no significant agreement (Fig 2A).

### Detection of *Fasciola* species using a newly developed qPCR assay

**Fig 3:**
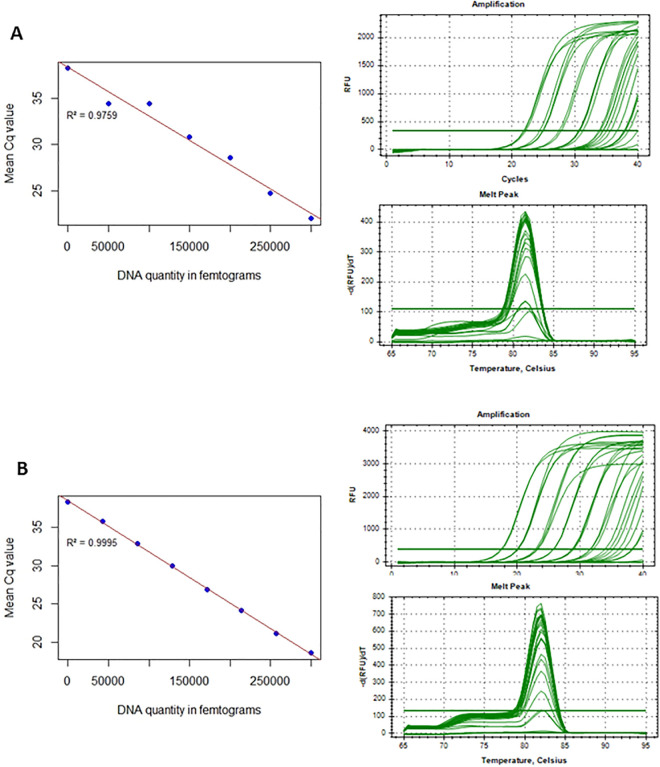
Sensitivity of qPCR targeting mt-ND1 mitochondrial DNA of (A) *F. hepatica* and (B) *F. gigantica.* The standard curve was plotted using qPCR data from 5-fold serial dilutions of adult worm DNA extracted from the head of *F. hepatica* and *F. gigantica*. DNA quantification was performed using Qubit. The average Cq values from three replicates were plotted against the DNA quantity to create the standard curve. The amplification profiles and melt curves for *F. hepatica* and *F. gigantica* at 81.5°C and 82°C respectively are also shown.

To provide a simple, low-cost, sensitive, universal and accurate molecular method for diagnosing *Fasciola* spp. infections in eggs sedimented from faeces, a SYBR green qPCR assay was developed using a repurposed primer set targeting mt-ND1 specific to *F. hepatica* and *F. gigantica.* The assay’s analytical sensitivity was first assessed using quantified known *F. hepatica* and *F. gigantica* adult worm DNA (positive controls). The assay’s limit of detection based on a 1:5 DNA dilution series was found to be 19.2 fg for *F. hepatica* and 6.4 fg for *F. gigantica* DNA. A linear standard curve was generated, showing an efficiency of 97% (R² = 0.9759) for *F. hepatica* and 99% (R² = 0.9995) for *F. gigantica,* demonstrating efficient primer binding and target amplification. Additionally, qPCR melt curve analysis identified a distinct peak at 81.50°C for *F. hepatica* and 82°C for *F. gigantica* (Fig 3A and B), confirming the specificity of primer binding to the DNA target, and absence of nonspecific primer interactions.

**Fig 4:**
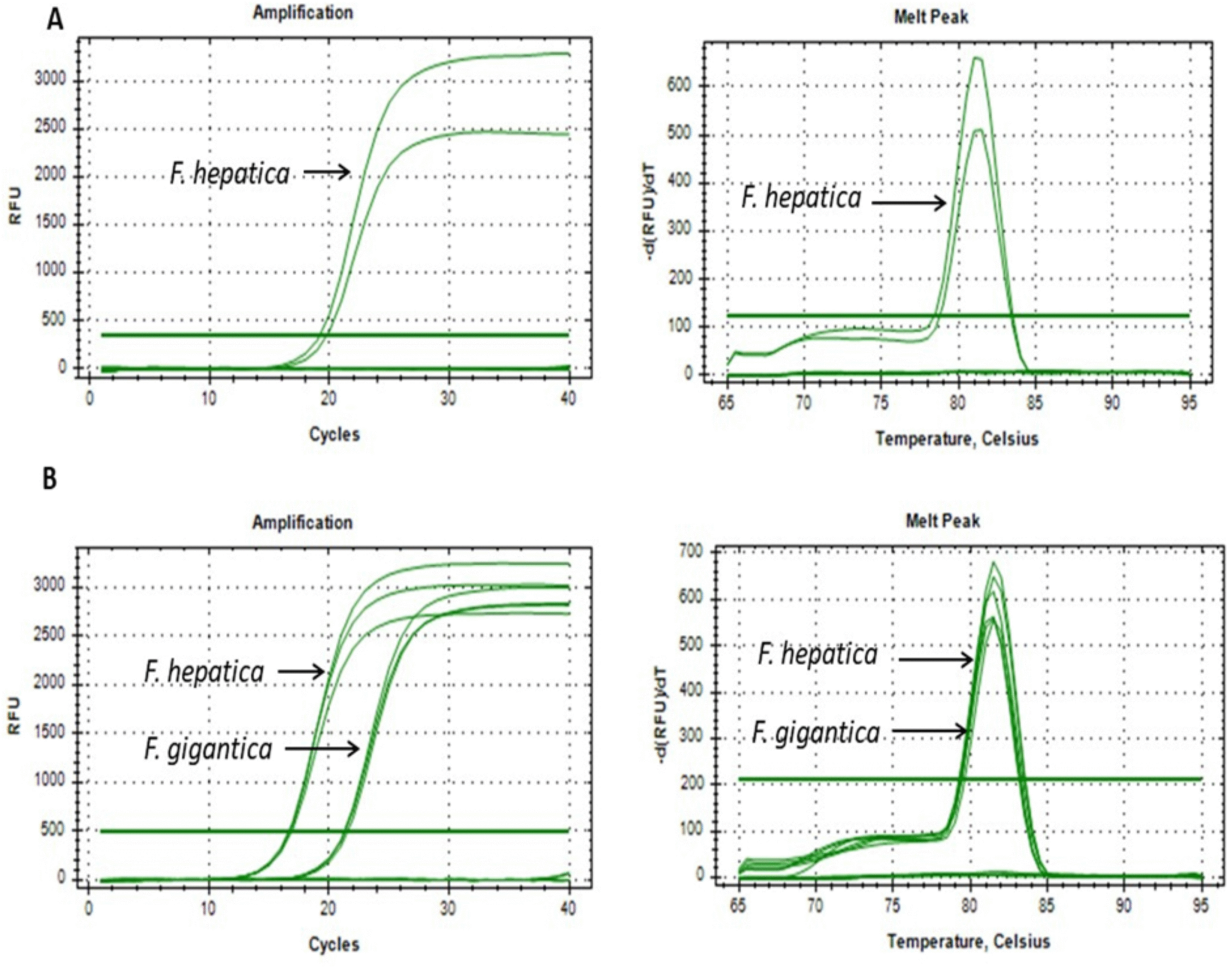
Specificity of mt-ND1 primers in qPCR for amplification of *F. hepatica* and *F. gigantica.* **(A)** Specificity of mt-ND1 primers evaluated with DNA of other flukes, *Paramphistomum*, *E.* e*xplanatum*, and *C. daubneyi.* **(B)** Specificity of mt-ND1 primers tested with DNA of the nematode, *T. circumcincta*. Amplification curves are shown on the left-hand side and melt curves on the right-hand side. There is no cross-amplification with non-target species in any of the reactions.

The amplified mt-ND1 fragment is highly conserved between *F. hepatica* and *F. gigantica.* As a result, the melt curve analysis can confirm the presence of *Fasciola* spp. but is not able to distinguish clearly between the two species. The specificity of the qPCR assay was evaluated against DNA from other prevalent fluke and nematode species. The melt curve analysis confirmed that only *F. hepatica* and *F. gigantica* produced amplification peaks at 81.50°C and 82°C, respectively, with no cross-amplification of other fluke species (Fig 4). The assay exhibited strong reproducibility, with coefficients of variation (CV) below 6.0% for intra-assay and inter-assay. The mean Cq values ranged from 21.96 to 38.26 for *F.* hepatica with DNA dilutions ranging from 300 pg to 19.2 fg and 18.62 to 38.24 for *F. gigantica* with DNA dilutions ranging from 500 pg to 6.4 fg, maintaining consistency across replicates (Table 2).

**Table 2:** Variation in *F. hepatica and F. gigantica* SYBR green qPCR Cq values within and between assays using data from the test of analytical sensitivity.

DNA conc.	Cq values observed for *Fasciola hepatica* DNA				Cq values observed for *Fasciola gigantica* DNA		
	Mean*	SD	CV%	DNA conc.	Mean	SD	CV%
300 pg	21.96	0.16	0.72	500 pg	18.62	0.147	0.78
60 pg	24.73	0.327	1.32	100 pg	21.13	0.066	0.31
12 pg	28.59	1.717	6.00	20 pg	24.16	0.195	0.80
2.4 pg	30.84	0.347	1.12	4 pg	26.85	0.023	0.08
480 fg	34.46	0.330	0.95	800 fg	29.94	0.142	0.47
96 fg	34.43	0.602	1.74	160 fg	32.91	0.29	0.88
19.2 fg	38.26	0.343	0.89	32fg	35.73	0.614	1.71
				6.4 fg	38.24	1.063	2.77

* Mean of 3 replicates; Cq: PCR cycle number, SD: standard deviation, CV: coefficient of variation.

### Fluke species detection using microscopy and qPCR

To gain further clarification on which fluke species were present in the 128 field samples identified as egg positive by microscopy, the newly developed SYBR green qPCR was utilised. In total 123 of the 128 egg-positive samples were tested by qPCR as DNA was not available or DNA extraction failed for five samples. Overall, 57 samples (46.35%) were positive for *Fasciola* spp. by qPCR and 66 (53.65%) were negative. Of the 30 samples tested that were egg-negative by microscopy, one was found to be positive by qPCR.

**Fig 5:**
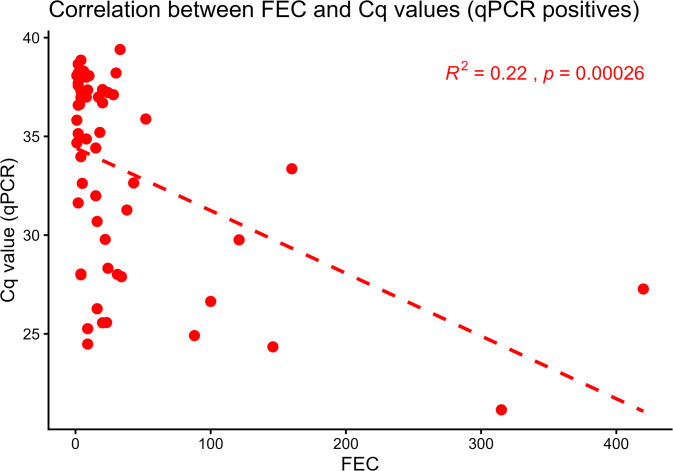
Correlation of fluke egg count (FEC) per gram of faecal material with Cq values observed in qPCR from natural *Fasciola* infections. A scatterplot of qPCR-positive samples is shown as red circles. The dotted red line indicates a simple linear regression analysis. Significant correlation (*p* = 0.00026, R^2^ = 0.22) was noted for between egg counts and Cq values, with higher egg counts was associated with lower Cq values.

Of the 71 samples identified as *F. hepatica* positive by microscopy (EPG range: 1–315, SE: 5.87), 67 samples (EPG range: 1–315, SE: 6.07) were subjected to qPCR, as four samples were not screened due to failed DNA extraction or insufficient DNA sample. The qPCR confirmed *F. hepatica* in 34 (50.75%) samples (EPG range: 1–315, SE: 9.87), while 33 (49.25%) samples (EPG range: 1–170, SE: 7.11) were negative. Of the 30 samples identified as *C. daubneyi* positive by microscopy (EPG range: 1–60, SE: 2.27), qPCR detected *F. hepatica* in seven (23.33%) samples (EPG range: 1–28, SE: 3.68), whereas 23 (76.67%) samples (EPG range: 1–60, SE: 2.76) tested negative. Of the five samples identified as mixed infections by microscopy (EPG range: 52–160, SE: 23.61), qPCR confirmed four (80%) as positive for *F. hepatica* infection (EPG range: 52–160, SE: 23.05), while one (20%) sample (EPG: 28) tested negative. Finally, of the 22 samples that were unconfirmed using microscopy (EPG range: 1–420, SE: 18.89), 21 (95.45%) were screened by qPCR as DNA extraction was failed for 1 sample. The qPCR identified *F. hepatica* in 12 samples (57.15%) (EPG range: 1–420, SE: 34.71) (Table 3, S6 Table). The Cohen’s kappa coefficient (κ = 0.16, Z = 2.47, p = 0.013) showed low agreement between microscopy and qPCR for identification of *F. hepatica*, with an overall confusion matrix accuracy of 49.6% (95% CI: 40.5–58.8%). The ROC curve for *F. hepatica* showed an AUC of 0.58 (95% CI: 0.49–0.66), indicating poor agreement between microscopy and qPCR (Fig 2B). However, a significant correlation was observed between FEC and qPCR Cq values across all samples positive for *F. hepatica* by microscopy (p = 0.00026, R^2^ = 0.22) (Fig 5).

**Table 3:** Fluke species identification by microscopy, qPCR (for *F. hepatica*), and deep amplicon sequencing on 128 fluke egg-positive samples.

Microscopy positive	qPCR					Deep amplicon sequencing					
	Negative	Positive	ND^2^	NP	Total	*Fasciola hepatica*	*Calicophoron daubneyi*	Mixed	ND	No reads	Total
*Fasciola hepatica*	33	34	2	2	71	11	26	30	2	2	71
*Calicophoron daubneyi*	23	7	0	0	30	2	21	7	0	0	30
Mixed	1	4	0	0	5	0	1	4	0	0	5
Fluke^1^	9	12	1	0	22	0	3	17	1	1	22
Total	66	57	3	2	128	13	51	58	3	3	128

^1^Unidentified flukes; ^2^ND = DNA extraction failed; NP=Not performed.

### Detection of fluke species using a deep amplicon sequencing method

To accurately identify mixed-species as well as single-species fluke infections, a deep amplicon sequencing assay was developed using a universal primer set targeting the rDNA ITS2 region, which is specific to fluke species. From this region of rDNA, a variation in genetic identity was observed between different fluke species, ranging from 40% to 99% (S5 Table). A phylogenetic tree of 154 unique sequences showed distinct clustering of each fluke species (S1 Fig).

**Fig 6:**
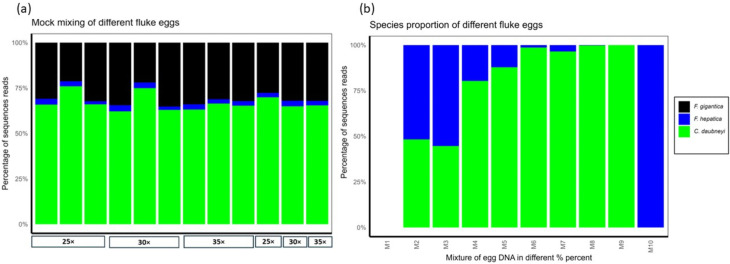
Sequence representation of the mock mixture of fluke species in deep amplicon sequencing. **(a)** *F. hepatica*, *F. gigantica*, and *C. daubneyi*. DNA was extracted in triplicate from pooled samples containing 250 eggs of each species. The DNA mixture was amplified using PCR with different numbers of cycles (25 ×, 30 ×, and 35×), with triplicate testing for each pool. The x-axis indicates PCR cycle number, while the y-axis represents the percentage of ITS2 rDNA sequence reads for each species. Triplicate runs were grouped based on the number of amplification cycles and averages are displayed in the last three columns. **(b)** Relative proportions of *F. hepatica* and *C. daubneyi* in egg DNA mixtures were assessed using deep amplicon sequencing. DNA was extracted from mock pools containing varying ratios of these two fluke species, enabling evaluation of the assay’s accuracy across a range of species mixes. The x-axis represents egg mixtures with varying *F. hepatica*: *C. daubneyi* ratios: M1 (negative control), M2 (99:1), M3 (90:10), M4 (70:30), M5 (50:50), M6 (30:70), M7 (10:90), M8 (1:99), M9 (100% *C. daubneyi*), and M10 (100% *F. hepatica*). The y-axis shows the percentage of ITS2 rDNA sequence reads for each species.

Initially, the sequence representation of three different fluke species *F. hepatica*, *F. gigantica* and *C. daubneyi,* in the deep amplicon sequencing assay was determined (Fig 6a). This allowed the evaluation of DNA sequence representation in output reads from samples with known species ratios. Each species showed consistent representation across triplicates in the sequence counts for each mixture (Fig 6a). Specifically, we observed that *C. daubneyi* consistently generated disproportionately higher reads, indicating biases in species representation (Fig 6a and 6b, and S1 File). Furthermore, the number of cycles (25 ×, 30× and 35×) used during the adaptor PCR were validated to ensure sufficient DNA was generated for sequencing whilst maintaining a balance between amplification efficiency and accuracy. Whilst it is known that an appropriate number of cycles helps minimise deviations and PCR bias, and over-amplification causing sequence dominance, we found that the number of cycles did not affect the sequence representation of any species (Fig 6a) [49]. In each mock pool, *C. daubneyi* generated the highest number of sequence reads, followed by *F. gigantica* and *F. hepatica*. Despite these trends, no statistically significant differences were observed in the representation of any species across the different PCR amplification cycles (*F. gigantica*, *P* = 0.730; *F. hepatica*, *P* = 0.774; and *C. daubneyi*, *P* = 0.258) (Fig 6a).

**Fig 7:**
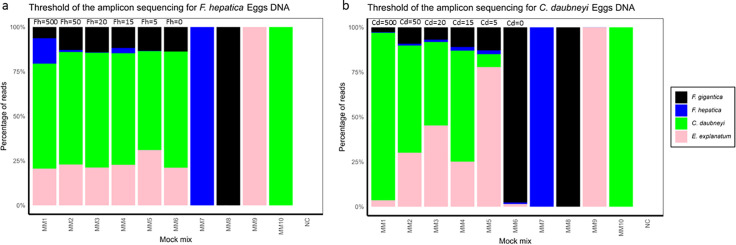
Threshold of deep amplicon sequencing. Application of deep amplicon sequencing was assessed in mock egg mixtures with gradually decreasing counts of *F. hepatica* and *C. daubneyi* eggs. Two sets of mixtures were designed using eggs from four fluke species (*F. hepatica*, *F. gigantica*, *E. explanatum* and *C. daubneyi*). In panel **(a)**, which focuses on *F. hepatica*, MM1 contained 500 eggs of *F. hepatica* along with 50 eggs of each of the other three species, creating a high relative abundance of *F. hepatica*. In mixtures MM2 through MM6, the number of *F. hepatica* eggs was reduced to 50, 20, 15, 5 and 0 eggs, respectively, while the counts for the other three fluke species remained constant at 50 eggs. Panel (b) follows a similar design but targets *C. daubneyi*: MM1 contained 500 eggs of *C. daubneyi* plus 50 eggs each of *F. hepatica*, *F. gigantica*, and *E. explanatum*, and in mixtures MM2 to MM6 the number of *C. daubneyi* eggs was reduced to 50, 20, 15, 5 and 0 eggs, with the other species maintained at 50 eggs each. Additionally, single-species control pools were included as MM7 (*F. hepatica*), MM8 (*F. gigantica*), MM9 (*E. explanatum*), and MM10 (*C. daubneyi*), each containing 50 eggs. The assay results show the ability to detect and accurately quantify trace levels of target DNA in mixed fluke egg populations.

We further assessed the assay’s accuracy in identifying relative species proportions in mixed infections by testing pairwise mixtures of *F. hepatica* and *C. daubneyi* eggs (Fig 6b). This range of mixes enabled thorough validation of the sequencing assay, demonstrating we could detect both fluke species across egg ratios, however, the proportion of reads did not always match the egg ratios. These species were selected due to their high prevalence, frequent co-occurrence in UK cattle and sheep herds, and availability in our laboratory (Fig 6b, S2 File). This approach addressed the sensitivity of deep amplicon sequencing assays in detecting trace-level amplicons. We observed variations in species sequence read representation across different mixes, which may affect the overall interpretation of relative species abundance. For example, the 99% *F. hepatica:* 1% *C. daubneyi* mix M2 and the 90% *F. hepatica:* 10% *C. daubneyi* mix M3 displayed similar species representation (Fig 6b). Further, we tested the thresholds of the deep amplicon assay for fluke egg DNA detection with decreasing egg levels in mixed populations of *F. hepatica* and *C. daubneyi* (Figs 7a and 9b, S3 File and S4 File). A notable observation was the production of a lower number of sequenced reads, particularly for *F. hepatica* (Fig 7a and 7b); however, this did not affect the species identification. Importantly, the assay detected both *F. hepatica* and *C. daubneyi* DNA at levels down to 5 eggs per pool.

**Fig 8:**
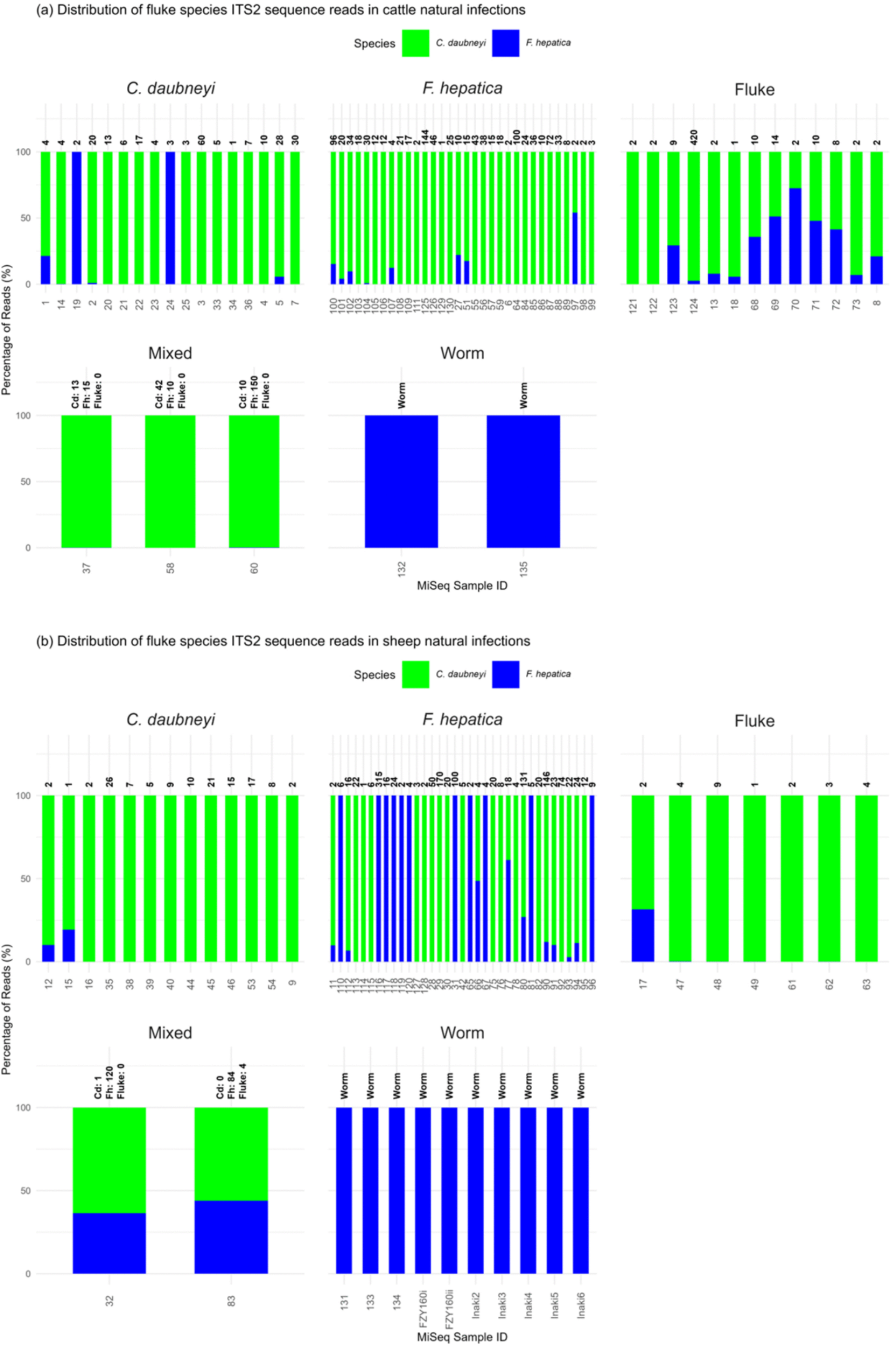
Deep amplicon sequencing applied to field samples. This figure illustrates the application of the deep amplicon sequencing assay on DNA extracted from sedimented faecal eggs and adult worm populations collected from cattle and sheep across various regions in the UK. The charts displays five groups based morphological identification by microscopy including *C. daubneyi*, *F. hepatica*, fluke (unspeciated samples), mixed eggs and worms (adult *F. hepatica* worms). Eggs per gram for each sample is shown above the bars. Percentage bars show species sequence reads obtained from deep sequencing generated after 35 amplification cycles. *F. hepatica* read percentages are represented in blue and *C. daubneyi* in green. **(a)** Samples from cattle. **(b)** Samples from sheep.

After validation, the assay was applied to 125 of the 128 fluke egg-positive samples to analyse fluke species distributions in natural infections in cattle and sheep (Fig 1b), as well as DNA from adult fluke. Out of the 125 faecal samples sequenced, 122 (97.6%) produced reads and 3 samples (2.4%) failed to yield sequencing reads. In cattle, 67 samples (eggs: n = 65, worms: n = 2) produced sequence reads (Fig 8a, S5 File). The sequence reads data revealed that *F. hepatica* was present in 4 samples (eggs: n = 2, worms: n = 2) (sequence reads range: 203–3492, average reads:1628), *C. daubneyi* in 28 samples (sequence reads range: 3614,691, average reads: 5985), and mixed infections in 35 faecal samples (sequence reads range: 369–13,138, average reads: 5354). Similarly, in sheep, 67 samples (eggs: n = 57, worms: n = 10) generated sequence reads (Fig 8b, S5 File). The data showed *F. hepatica* in 21 samples (eggs: n = 11, worms: n = 10) (sequence reads range: 13–85,233, average reads: 21,752), *C. daubneyi* in 23 samples (sequence reads range: 50–14,083, average reads = 6069), and mixed infections in 23 faecal samples (sequence reads range = 367–19,257, average reads = 6218). This analysis highlights a higher prevalence of mixed infections followed by *C. daubneyi* and *F. hepatica* single infections in cattle and sheep. The sequence reads of all samples were aligned with *F. hepatica* and *C. daubneyi* using BLASTn (https://data.mendeley.com/datasets/zyvwc6ppy8/2).

**Fig 9:**
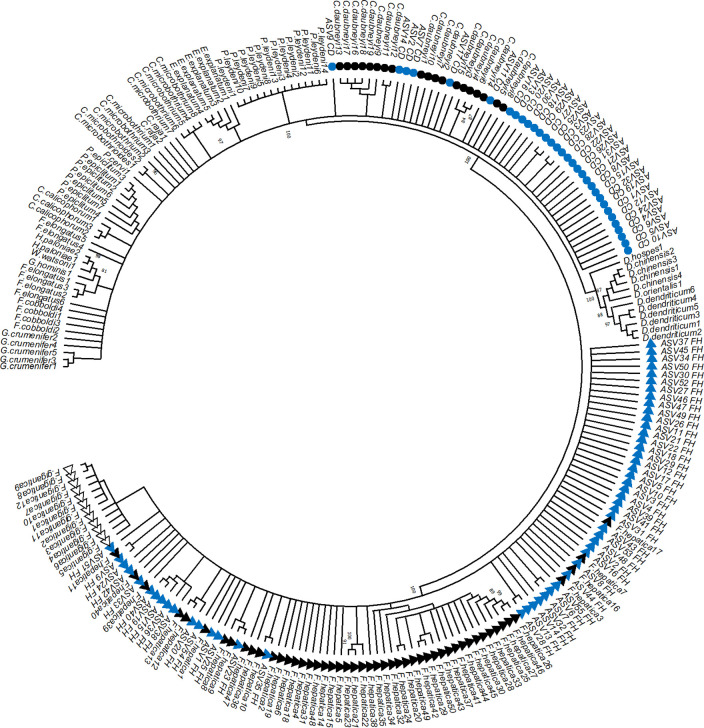
Neighbour-joining tree of rDNA ITS2 sequences constructed using 154 reference sequences of different fluke species downloaded from the NCBI database, along with 87 ASVs identified in this study, 55 from *F. hepatica* and 32 from *C. daubneyi.* ASVs corresponding to *F. hepatica* are marked with blue triangles, while those of *C. daubneyi* are represented by blue circles. The black triangles indicate *F. hepatica,* white triangles indicate *F. gigantica* and black circles indicate *C. daubneyi* sequences from the NCBI database. The ASVs clustered closely with their respective reference taxa, confirming accurate taxonomic assignment.

Finally, ASVs were generated for *C. daubneyi* and *F. hepatica* from all sequence reads of the samples collected from different counties in the UK. ASVs were up to 461 bp and 527 bp for *C. daubneyi* and *F. hepatica*, respectively. In total, 87 ASVs were identified, including *F. hepatica* (n = 55) and *C. daubneyi* (n = 32) (https://data.mendeley.com/datasets/zyvwc6ppy8/2). A phylogenetic tree of all ASVs with reference sequences of 21 fluke species showed that *F. hepatica* and *C. daubneyi* species separated into distinct clades (Fig 9).

### Microscopy and deep amplicon sequencing for fluke species detection

Of the 71 samples (EPG range: 1–315, SE: 5.87) identified as *F. hepatica* by microscopy, deep amplicon sequencing detected only 11 (EPG range: 2–315, SE: 28.39) as single *F. hepatica* infections. However, deep amplicon sequencing classified 26 of the 71 samples as *C. daubneyi* (EPG range: 1–170, SE: 8.45) and 30 as mixed infections (EPG range: 2–96, SE: 5.63). Among the 30 samples marked as *C. daubneyi* by microscopy (EPG range: 1–60, SE: 2.27), deep amplicon sequencing detected two as *F. hepatica* (EPG range: 2–3, SE: 0.5), 21 as *C. daubneyi* (EPG range: 1–30, SE: 1.75), and seven as mixed infections (EPG range: 1–60, SE: 8.16). For the microscopically recognised five mixed infections (EPG range: 52–160, SE: 23.61), deep amplicon sequencing confirmed four as mixed infections (EPG range: 28–160, SE: 27.93), while one was classified as *C. daubneyi* (EPG range: 52). Lastly, among the 22 samples that were undecided by microscopy (EPG range: 1–420, SE: 18.89), deep amplicon sequencing identified three as *C. daubneyi* (EPG range: 2–4, SE: 0.66), 17 as mixed infections (EPG range: 1–420, SE: 24.42), and two as not determined or with failed DNA extraction (EPG range: 4–5, SE: 0.5) (Table 3, S6 Table). Cohen’s kappa = 0.14, (Z = 4.06, p < 0.0001) showed a low level of agreement between microscopy and deep amplicon sequencing in identification of fluke species. The ROC curve also indicated low agreement of two methods for identifying *F.* *hepatica* (AUC = 0.67, 95% CI: 0.55–0.78), rumen fluke (AUC = 0.64, 95% CI: 0.56–0.72), and for mixed infections (AUC = 0.53, 95% CI: 0.49–0.56) (Fig 2C).

### qPCR and deep amplicon sequencing for fluke species detection

There were 20 samples (EPG range: 2–74, SE: 4.39) that tested negative by qPCR; however, *F. hepatica* sequences were detected by deep amplicon sequencing (three single and 17 mixed infections). Conversely, deep amplicon sequencing did not produce *F. hepatica* reads for eight qPCR-positive samples (EPG range: 4–52, SE: 6.52) (Table 4, S6 Table). However, due to mixed infections of *F. hepatica* and *C. daubneyi*, the egg counts for individual fluke species are not clear*.* Interestingly, *C. daubneyi* reads were generated for the eight samples, which were positive by *Fasciola* qPCR (mean Cq range: 31.27-37.37), but did not have *F. hepatica* reads in deep amplicon sequencing.

**Table 4:** Fluke species identification by qPCR and deep amplicon sequencing on 128 fluke egg-positive samples.

qPCR	Deep amplicon sequencing					
	*Calicophoron daubneyi*	*Fasciola hepatica*	Mixed	ND	No reads	Total
Negative	43	3	17	0	3	66
*Fasciola hepatica*	8	10	39	0	0	57
ND	0	0	0	3	0	3
NP	0	0	2	0	0	2
Total	51	13	58	3	3	128

ND = DNA extraction failed, NP =Not performed.

There was a good agreement with qPCR for samples which were *F. hepatica* positive by deep amplicon sequencing based on the ROC curve AUC = 0.77 (95% CI: 0.70–0.84) (Fig 2D). Cohen’s kappa of 0.54 (Z = 5.99, p < 0.001) indicated moderate agreement between methods with a confusion matrix accuracy of 76.7% (95% CI: 68.1–84.0%). Further, a significant association (*p* < 0.001) was noted between the identification of *F. hepatica* infections using qPCR and the deep amplicon sequencing approach using Fisher exact test.

### Fluke species identification using Sanger sequencing and deep amplicon sequencing

Sanger sequencing identified 11 samples as *F. hepatica,* of which deep amplicon sequencing confirmed six (54.54%) as *F. hepatica*, and five (45.45%) as mixed infections, and none as *C. daubneyi*. Among the 65 samples identified as *C. daubneyi* by Sanger sequencing, deep amplicon sequencing confirmed 36 (55.38%) as *C. daubneyi* and 29 (44.62%) as mixed infections. Notably, the sample identified as *P. epiclitum* by Sanger sequencing was detected as *F. hepatica* in deep amplicon sequencing (Table 5, S6 Table).

**Table 5:** Fluke species identification by Sanger sequencing and deep amplicon sequencing on 128 fluke egg-positive samples.

Deep amplicon sequencing	Sanger sequencing using ITS2						
	*Fasciola hepatica*	*Calicophoron daubneyi*	*Paramphistomum epiclitum*	PSQ	Not performed	ND^1^	Total
*Fasciola hepatica*	6	0	1	6	0	0	13
*Calicophoron daubneyi*	0	36	0	15	0	0	51
Mixed	5	29	0	19	5	0	58
No reads	0	0	0	3	0	0	3
ND	0	0	0	0	0	3	3
Total	11	65	1	43	5	3	128

^1^ND = DNA extraction failed; PSQ = poor sequence quality.

The ROC curve analysis indicated a good agreement between deep amplicon sequencing and Sanger sequencing identification of *F. hepatica* (AUC = 0.77, 95% CI: 0.61–0.92) and rumen fluke (*C. daubneyi* plus *P. epiclitum*) (AUC = 0.77, 95% CI: 0.71–0.83). The ROC analysis could not be performed for the mixed infection, as ITS2 based Sanger sequencing could not classify samples as mixed infections (Fig 2E). Overall confusion matrix stats showed accuracy of 54.5% (95% CI: 42.8–65.9%), and Cohen’s kappa agreement between the two methods was fair (κ = 0.225, p < 0.001).

## Discussion

In this study, we present approaches for detecting and identifying fluke species in material sedimented from ruminant faecal samples in the form of qPCR with high analytical sensitivity and specificity for the detection of *Fasciola* spp. and a deep amplicon sequencing assay, which can accurately identify and differentiate between closely related fluke species, such as *F. hepatica*, *F. gigantica*, and *C. daubneyi*. These methods provide additional tools for fluke species identification beyond existing microscopy and molecular approaches. We selected the ITS2 and mt-ND1 genetic markers based on their previous application in fluke species identification and their potential to differentiate between closely related flukes [8,57].

Multiple direct and indirect diagnostic approaches are available for diagnosing fluke infections, each with its own set of limitations. Historically, the most widely used traditional direct identification method is detecting fluke eggs in the host’s faeces. This can be accomplished through various techniques such as FLOTAC [72], sedimentation, Flukefinder or the Kato-Katz thick smear method [73]. Whilst the use of fluke egg counts is simple, this diagnostic route can be unreliable due to low intermittent egg deposition in the faeces [74], is time-consuming and requires highly trained laboratory staff [3].

A number of molecular-level diagnostic methods based on DNA detection have been developed, including PCR techniques [37], qPCR [3,36], and LAMP approaches [38]. High-throughput deep amplicon sequencing of metabarcoded DNA from parasite populations using the Illumina MiSeq platform can offer a low-cost and potentially more accurate alternative to traditional microscopic methods. For instance, adult *Fasciola* spp. and *C. daubneyi* flukes have previously been detected using deep amplicon sequencing [8,50,51], and this method has been applied to field samples for the speciation of *F. hepatica* and *F. gigantica* in areas where they overlap [52].

In the present study, species identification through microscopic examination was first checked by PCR followed by Sanger sequencing. Approximately half of the microscopy-positive samples were confirmed by ITS2 Sanger sequencing to contain *F. hepatica* or *C. daubneyi* DNA. [12,31]. Although PCR bands were observed on the agarose gel for most of the samples, Sanger sequencing produced poor-quality sequence reads for many samples. Poor sequencing quality can be due to non-specific amplifications, artefacts, small amounts of PCR product for sequencing, and samples containing mixed DNA templates from double infections [75]. These findings indicated that Sanger sequencing has limitations in generating reliable sequence reads [75] for fluke speciation, particularly in complex natural infections.

We repurposed mt-ND1 markers to develop a SYBR Green qPCR assay to detect *Fasciola* spp. The choice to use SYBR Green over fluorescence probe-based systems was due to its cost-effectiveness, whereas previous studies have used TaqMan probes to identify *Fasciola* species [3,36,76]. Our assay’s analytical sensitivity when tested on purified genomic DNA was 19.2 fg for *F. hepatica* and 6.4 fg for *F. gigantica* DNA, although this does not accurately represent the the analytical sensitivity of the assay when applied to sedimented faeces. Similarly, previous studies have demonstrated the ability to detect *F. hepatica* at levels below 10 eggs per gram directly from 150 mg of faecal material using a TaqMan qPCR assay [3] and sensitivities as low as 1 pg/μL [36] and 1.6 pg/μL when targeting the ITS1 region [77]. *F. hepatica* eDNA (14–50 fg) was detected in water samples with similar sensitivity to our assay [78]. In 2024, a qPCR assay was reported that could detect 10 fg of *Fasciola* DNA in water and one pg in human stool samples [79]. A limitation of our analysis is that we performed limit of detection (LOD) studies in molecular-grade water rather than a more complex matrix such as faeces where inhibitors and DNA degradation could impact the assay. Future experiments using DNA-spiked faecal suspension could provide a more realistic estimate of LOD.

Overall, we observed a significant correlation between fluke egg counts and qPCR Cq values (R^2^ = 0.22), which showed a general relationship of higher egg counts resulting in lower Cq values [80], although the R^2^ value was lower than in previously published qPCR assays [3,81]. A limitation of our study is that we did not validate the qPCR against an established probe-based qPCR assay [3,36] or coproantigen ELISA [82]. In this study, not all negative samples were tested, as a consequence, it was not possible to statistically determine diagnostic performance. This will be determined in future work with a larger sample set of positive and negative samples to enable diagnostic sensitivity evaluation.

Another limitation of our qPCR assay is that we detected *Fasciola* in DNA extracted from sedimented material, rather than in DNA extracted directly from faecal samples, as in a number of other studies [3,12,76,83]. A handful of studies have applied molecular techniques to detect natural *Fasciola* infections directly from faecal material and reported limitations [3,83]. A recent study successfully detected *F. hepatica* in DNA extracted directly from faeces with a commercial kit, using both LAMP and PCR methodology targeting ITS2 region [84]. Further work is needed to simplify extraction protocols so that they are more field-friendly.

In ruminants, fluke species often occur in complex and overlapping infections; for instance, *F. hepatica* and *C. daubneyi* co-infections have been observed in cattle and sheep in the UK [85,86] and the same has been reported elsewhere in Europe [12,87], including in Ireland [88] and Germany [89]. We found that the microscopic egg identification for flukes, such as *F.* *hepatica* and *C. daubneyi*, is challenging due to their similar size and shape. Therefore, the deep amplicon sequencing method was developed utilising universal ITS2 rDNA markers to differentiate between multiple fluke species. The techniques were validated using fluke egg DNA, isolated from faecal samples. We used the deep amplicon sequencing approach to detect mixed fluke infections in material sedimented from faeces of naturally infected animals. Previously, the approach was applied to adult fluke samples [50,51] and the eggs of *Fasciola* species and their hybrids in faecal material [52]. In the current study, ITS2 deep amplicon sequencing provided species-level fluke differentiation, specifically between *F. hepatica* and *C. daubneyi*.

Deep amplicon sequencing generated sequence reads for *F. hepatica*, *F. gigantica*, and *C. daubneyi*. However, the proportion of sequence reads deviated from expected percentages. When we evaluated the assay’s ability to accurately determine the relative proportions of *F. hepatica* and *C. daubneyi* in pairwise combinations, a consistently higher proportion of reads were found for *C. daubneyi* compared to *F. hepatica* across all mixtures. Such variation may arise from factors including the primers used for the target loci, conserved priming sites, variations in DNA template concentrations during sample handling, the number of PCR cycles, and the copy number of the target DNA locus [52,90]. Previously, for nematodes, species-specific representation biases were addressed by calculating correction factors using L3 larval population DNA from different nematode species [43]. In the present study, calculation of correction factors did not remove sequence biases (S1 File). This might be due to differences in the eggshell chemistry or stability hardness of eggshells that has been described between *F. hepatica* and *C. daubneyi* [91], leading to variations in DNA extraction efficiency. However, we employed mechanical disruption before DNA isolation to mitigate this issue [3]. Additionally, we used universal ITS2 primers to detect multiple fluke species in a single deep amplicon sequencing run. Though it will increase workflow complexity, using species-specific primers in deep amplicon sequencing could be a potential solution to reduce sequence biases [92]. Further, bioinformatics analysis of sequencing data might introduce biases, leading to inaccuracies in species proportion estimations. One major challenge can be the limited availability of reference sequence reads in the NCBI database, for example, for *F. hepatica* 50 unique sequences and for *C. daubneyi* 19 unique sequences were found, which can impact species specific reads identification. Additionally, taxa represented by low numbers of sequencing reads may pose a problem, as these low-frequency reads were removed during data filtering while eliminating artefacts, resulting in the underrepresentation of actual sequence reads.

When applying the deep amplicon sequencing approach to field samples, just over half of the microscopy positive *F. hepatica* samples were confirmed by deep amplicon sequencing. Moreover, a significant correlation was found between the identification of *F. hepatica* infections using qPCR and deep amplicon sequencing. Using the deep amplicon sequencing approach, many *F. hepatica* and *C. daubneyi* co-infections were identified in field samples. Since *F. hepatica* is more pathogenic and economically detrimental [18,93] than *C. daubneyi* [94,95], and treatment choices differ, our method provides a valuable tool for identifying co-infections of these two significant parasites using faecal egg samples.

It should be noted that in some instances conflicting species identifications were observed using the different molecular methods. For example, some discrepancies were evident between Sanger sequencing and deep amplicon sequencing results. Most of these inconsistencies occurred where Sanger sequencing identified a single species (*F. hepatica* or *C. daubneyi*), whereas deep amplicon sequencing revealed mixed infections. Approximately half of the samples that produced poor-quality sequences by Sanger sequencing were identified as mixed infections using deep amplicon sequencing. The remaining poor-quality samples were mainly identified as *C. daubneyi*, with some identified as *F. hepatica*. Other inconsistencies between Sanger sequencing and deep amplicon sequencing results were likely due to reduced Sanger sequencing assay sensitivity, primer biases, or variable DNA template quality [67]. Furthermore, one sample identified as *P. epiclitum* by Sanger sequencing was not confirmed by deep amplicon sequencing, which instead identified it as *F. hepatica*. This could be due to low-quality Sanger sequencing, which may lead to inaccurate blast results and misidentification as *P. epiclitum*. Previously, *P. leydeni* has been reported in sheep [96] and deer [97] in Ireland, but was not identified in the UK in our study. Deep amplicon sequencing generated *F. hepatica* sequence reads in some samples that tested negative by qPCR. Conversely, deep amplicon sequencing did not produce *F. hepatica* reads for a few qPCR-positive samples, which may be related to the bias of the deep sequencing approach to *C. daubneyi* over *F. hepatica*. These findings highlight the inherent limitations of each method; however, deep amplicon sequencing can reliably identify mixed infections, which is more difficult if not impossible using the other methods.

Regarding the implementation of these methods, microscopy is suitable option for analysing a small number of samples due to its low cost and accessibility, although, there are potential issues of misidentifications [3,12]. In particular, a high proportion of samples identified as positive for *F. hepatica* based on egg morphology were confirmed by deep amplicon sequencing to contain *C. daubneyi*. For medium to high sample volumes, our qPCR assay is suitable for the identification of *Fasciola* spp. infections at genus level only. In the case of high sample volumes, deep amplicon sequencing is a potentially more effective choice for species-level differentiation of different flukes, with the advantage of high throughput, as a single Illumina MiSeq run can process up to 384 samples simultaneously.

A limitation of this study was that no formal statistical power analysis was performed to justify the sample size. Instead, sample availability was based on veterinary practitioner and farmer participation. However, we recognise that larger sample sizes guided by formal statistical calculations, in a range of endemic regions, and in different host species and application on both positive and negative samples will be required to fully assess the utility of these methods and their diagnostic sensitivity and specificity. Although molecular tools, including qPCR and deep amplicon sequencing, provide advantages in terms of species resolution, they also have drawbacks. These include higher costs in case of low number of sample processing, the need for specialised equipment and trained personnel, and longer turnaround times.

The sequencing data generated from the set of natural field samples enhanced our understanding of genetic variation within fluke populations. Notably, using ITS2 markers, we observed more ASVs for *F. hepatica* than *C. daubneyi*, indicating possible greater genetic diversity within *F. hepatica* populations. [8,50,57,98–100]. The ITS2 rDNA provides useful taxonomic resolution [8,57], however, ideally at least one mitochondrial marker such as (mt-ND1 and mt-CO1) and one nuclear genetic markers are preferred for detailed genetic level studies [71]. Therefore, further investigations using mitochondrial mt-ND1 and nuclear genetic markers are required to understand the genetic diversity of these fluke populations, and this work is in progress.

Our results also have important implications for “One Health”. Although validated on cattle and sheep samples in this study, both the qPCR and deep amplicon sequencing methods have a strong potential for the *Fasciola* spp. detection in humans and the environment, particularly in endemic regions where there is a possibility of zoonotic transmission of *F. hepatica* and *F. gigantica*. *Fasciola* spp. eggs shed in livestock faeces are contaminating pastures and freshwater systems, resulting in sustained parasite transmission cycles and posing potential risks to human health in endemic regions [74,101,102]. Improved molecular surveillance of livestock and the environment not only strengthens animal health management but also helps to control human exposure risk. Therefore, application of these methods on human faecal samples and environmental samples could improve case detection, fluke species identification, and epidemiological understanding.

Future studies should focus on designing and validating broader primer sets targeting mt-ND1 loci to enable amplification across multiple fluke species with distinct melting temperature profiles for different flukes. This approach will enhance the diagnostic potential of qPCR and applicability of the mt-ND1 + Melt Curve Analysis method. Another important future direction for this work could be the integration of high-throughput molecular diagnostic tools with predictive analytics to support surveillance and control. Machine learning approaches could combine molecular outputs, such as qPCR Cq values and deep sequencing species profiles, with epidemiological and environmental variables (e.g., climate, grazing management, animal movement) to develop risk assessment tools [103,104]. Such models may enable prediction of fluke infection risk at farm or regional scales, guiding targeted interventions and supporting precision livestock management. This integration aligns with the One Health framework by transforming diagnostic data into actionable insights for both veterinary and public health contexts.

## Conclusion

In conclusion, this study presents the use of deep amplicon sequencing for detecting mixed infections of *F. hepatica* and *C. daubneyi* and SYBR green assay for detection of *Fasciola* infections. The deep amplicon sequencing was effective and the preferred approach for detecting mixed fluke infections, compared to other techniques utilised in this study, demonstrating a high frequency of *C. daubneyi* and *F. hepatica* co-infections in farmed ruminants in the UK. The methods were primarily validated using samples from natural infections, with DNA extracted from faecal sedimented eggs, which allowed an easy and non-invasive sampling approach at the farm level. Deep amplicon sequencing and qPCR can be promising tools to complement microscopy and other existing tools in fluke disease surveillance and control in livestock and humans.

## Supporting information

S1 Fig Neighbour-joining tree generated for 21 different fluke species. (PDF)

S1 File Correction factor calculations. (PDF)

S2 File Relative proportions of *F. hepatica* and *C. daubneyi* in egg DNA mixtures. (XLSX)

S3 File Mock egg mixtures with gradually decreasing counts of *F. hepatica* eggs. (XLSX)

S4 File Mock egg mixtures with gradually decreasing counts of *C. daubneyi* eggs. (XLSX)

S5 File Deep amplicon sequence reads generated from field samples from cattle and sheep. (XLSX)

S1 Table Sample information. (PDF)

S2 Table Reference sequences downloaded from NCBI and unique sequence count. (XLSX)

S3 Table mt-ND1 and ITS2 primer sequences. (PDF)

S4 Table Raw read counts and quality report for deep amplicon sequencing. (XLSX)

S5 Table Genetic distances for different fluke species based on the ITS2 marker. (CSV)

S6 Table Sample (n = 128) details used for comparison of techniques for microscopy, PCR, Sanger sequencing, qPCR, and deep amplicon sequencing. (XLSX)
