# Supplementary material for: Development of a qPCR assay for Fasciola spp. identification and a deep amplicon sequencing method for differentiation of fluke species in UK livestock
Source: PLoS Negl Trop Dis. 2026 Feb 17;20(2):e0014006. doi: 10.1371/journal.pntd.0014006 (PMC12928598; doi:10.1371/journal.pntd.0014006)
Supplement: S1 Fig — (PDF) [file pntd.0014006.s001.pdf]

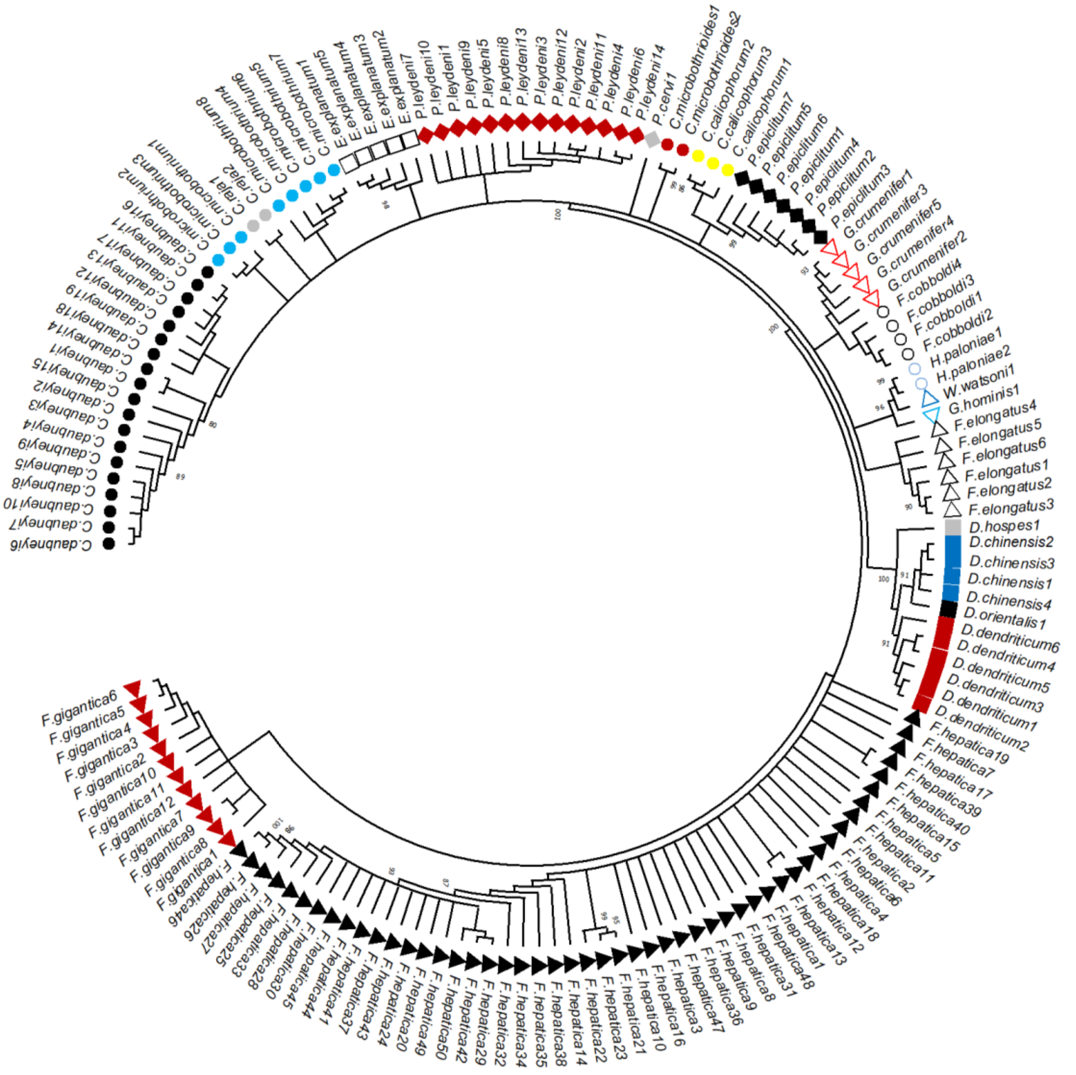

Fig. S1: Neighbour-joining tree generated for fluke species, including reference sequences of 21 different fluke species. The reference sequences were downloaded from NCBI, and 154 unique sequences were selected and aligned (Table S2, DOI: 10.17632/zyvwc6ppy8.1). Different fluke species are indicated with symbols of different colours and shapes. ***F. hepatica*** red triangle, ***F. gigantica*** black triangle, ***C. daubneyi*** black circle, ***Paramphistomum leydeni*** red diamond, ***P. cervi*** grey diamond, ***C. microbothrium*** light blue circle, ***P. epiclitum*** black diamond, ***Gastrothylax crumenifer*** triangle **with** red boundary no fill, ***Fischoederius elongatus*** triangle **with** black boundary no fill, ***Dicrocoelium dendriticum*** red **square**, ***C. calicophorum*** yellow circle, ***Fischoederius cobboldi*** circle with black boundary no fill, ***C. microbothrioides*** red circle, *Homalogaster* ***paloniae*** circle with light blue boundary no fill, ***E. explanatum*** **square** with black boundary no fill, ***C. raja*** grey circle, ***Watsonius watsoni*** triangle with dark blue border no fill, ***Gastrodiscoides hominis*** triangle with light blue boundary no fill, ***D. orientalis*** black **square**, ***D. hospes*** grey **square** and ***Dicrocoelium chinensis*** dark blue **square**.
