# Supplementary material for: Development of a qPCR assay for Fasciola spp. identification and a deep amplicon sequencing method for differentiation of fluke species in UK livestock
Source: PLoS Negl Trop Dis. 2026 Feb 17;20(2):e0014006. doi: 10.1371/journal.pntd.0014006 (PMC12928598; doi:10.1371/journal.pntd.0014006)
Supplement: S1 Table — (PDF) [file pntd.0014006.s007.pdf]

Table S1: Detailed information for the samples (n=402) collected between December 2022 and May 2024 by farmers and registered veterinary practitioners in the UK.

	Dec 2022 to June 2023					Sept 2023 to May 2024			
	**Cattle**	**Sheep**	**Alpaca**	**Goat**	**Water Buffalo**	**Cattle**	**Sheep**	**Host unknown**	**Total**
Pooled samples	10	44	0	2	0	33	68	0	157
Individual samples	46	46	4	0	1	54	70	0	221
No info about pooling or individual	0	0	0	0	0	11	5	8	24
Total samples	56	90	4	2	1	98	143	8	402
**Details of samples**									
History of fluke	38	38	0	2	0	42	71	0	191
No history of fluke	13	43	0	0	0	15	48	0	119
Unknown history of fluke	5	9	4	0	1	41	24	8	92
Total	56	90	4	2	1	98	143	8	402
