# Supplementary material for: Development of a qPCR assay for Fasciola spp. identification and a deep amplicon sequencing method for differentiation of fluke species in UK livestock
Source: PLoS Negl Trop Dis. 2026 Feb 17;20(2):e0014006. doi: 10.1371/journal.pntd.0014006 (PMC12928598; doi:10.1371/journal.pntd.0014006)
Supplement: S3 Table — (PDF) [file pntd.0014006.s009.pdf]

Table S3:

mt-ND1 and ITS2 primer sequences for amplifying mitochondrial region NADH dehydrogenase 1 and internal transcribed spacer 2 region of the rDNA respectively. The adaptor sequences are in italics, Forward and reverse primers are underlined. The 'N's represent random nucleotides added between the Illumina adaptor sequences and the locus-specific primers. Modified phosphate bonds at positions indicated with asterisks

Primer ID	Target	Primer sequence	Product length (bps)	Remarks	Reference
mt-ND1	Mitochondrial markers	UN2 (For): 5'-GTTTAAGTTTGTGTTTTTTC-3'<br>ND1 (Rev): 5'-CACCATAACTCCCCCAAACCA-3'	311	Primers used in qPCR to amplify mitochondrial region NADH dehydrogenase 1	(Rehman et al., 2020)
ITS2	Coding regions of 5.8S and 28S rDNA	ITS2 (For): 5'-GGTGGATCACTCGGCTCGTG-3'<br>ITS2 (Rev): 5'-TTCCTCCGCTTAGTGATATGC-3'	490-743	Universal ITS2 region primers	(Chaudhry et al. 2016)
AD_For ITS2	ITS2 + adaptor primer	*TCGTCGGCAGCGTCAGATGTGTATAAGAGACAG*<br><u>GGTGGATCACTCGGCTCG*T*G</u>	490-743	Forward direction	This work
AD_For 1N ITS2	ITS2 + adaptor primer	*TCGTCGGCAGCGTCAGATGTGTATAAGAGACAGN*<br><u>GGTGGATCACTCGGCTCG*T*G</u>	490-743	Forward direction	This work
AD_For 2N ITS2	ITS2 + adaptor primer	*TCGTCGGCAGCGTCAGATGTGTATAAGAGACAGNN*<br><u>GGTGGATCACTCGGCTCG*T*G</u>	490-743	Forward direction	This work
AD_For 3N ITS2	ITS2 + adaptor primer	*TCGTCGGCAGCGTCAGATGTGTATAAGAGACAGNNN*<br><u>GGTGGATCACTCGGCTCG*T*G</u>	490-743	Forward direction	This work
AD_Rev ITS2	ITS2 + adaptor primer	*GTCTCGTGGGCTCGGAGATGTGTATAAGAGACAG*<br><u>TTCCTCCGCTTAGTGATAT*G*C</u>	490-743	Reverse direction	This work
AD_Rev 1N ITS2	ITS2 + adaptor primer	*GTCTCGTGGGCTCGGAGATGTGTATAAGAGACAGN*<br><u>TTCCTCCGCTTAGTGATAT*G*C</u>	490-743	Reverse direction	This work
AD_Rev 2N ITS2	ITS2 + adaptor primer	*GTCTCGTGGGCTCGGAGATGTGTATAAGAGACAGNN*<br><u>TTCCTCCGCTTAGTGATAT*G*C</u>	490-743	Reverse direction	This work
AD_Rev 3N ITS2	ITS2 + adaptor primer	*GTCTCGTGGGCTCGGAGATGTGTATAAGAGACAGNNN*<br><u>TTCCTCCGCTTAGTGATAT*G*C</u>	490-743	Reverse direction	This work
